# The Influence of Freeze-Dried Alcohol-Water Extracts from Common Yarrow (*Achillea millefolium* L.) and German Chamomile (*Matricaria chamomilla* L.) on the Properties of Elastomer Vulcanizates

**DOI:** 10.3390/ijms232315048

**Published:** 2022-11-30

**Authors:** Andrii Aleksieiev, Marcin Masłowski, Magdalena Efenberger-Szmechtyk, Krzysztof Strzelec

**Affiliations:** 1 Institute of Polymer & Dye Technology, Lodz University of Technology, Stefanowskiego 12/16, 90-924 Lodz, Poland; 2 Institute of Fermentation Technology & Microbiology, Lodz University of Technology, Wolczanska 171/173, 90-530 Lodz, Poland

**Keywords:** common yarrow, German chamomile, natural rubber, freeze-dried extracts, antioxidants, bio-additives, elastomer vulcanizates

## Abstract

This research work aimed to investigate the properties of freeze-dried extracts from *Matricaria chamomilla* L. and *Achillea millefolium* L. and to perform a characterization of their impact on the natural rubber-based vulcanizates. First, extracts were prepared in three different solvents at selected volume ratios: water (100), water-methanol (50/50), and water-ethanol (50/50). Next, the freeze-drying of extracts was established and then obtained bio-additives were introduced to the rubber mixtures. Freeze-dried extracts were investigated by UV-VIS diffuse reflectance spectroscopy, Fourier Transform Infrared Spectroscopy (FTIR), Near-Infrared spectroscopy (NIR) and thermogravimetric analysis (TGA). Antioxidant activity and total phenolic content (TPC) were also defined. Rubber mixtures were examined in a rheometer and after vulcanization they were subjected to accelerated simulated aging by UV radiation and thermo-oxidative aging. To determine the resistance of vulcanizates to the degradation processes, the study of cross-linking density (equilibrium swelling method), mechanical properties (tensile strength, elongation at break) and color change were conducted. Performed studies proved the antioxidant activity of freeze-dried extracts caused by the high content of polyphenols and their beneficial influence on the properties of elastomer vulcanizates.

## 1. Introduction

*Achillea millefolium* L., commonly referred to as common yarrow, is a herbal plant from the Asteraceae family growing wild in the Northern hemisphere mainly in North America and Eurasia [1]. It naturally occurs along highways, pastures and fields, and it flowers twice a year from May to September [2]. Common yarrow is a perennial herb with hard and rhizomatous stems. It is characteristic of its abundant leaves and long, pinnate flowers, which may be white or pink. 

According to the literature, yarrow is a biologically active plant, which demonstrates multiple beneficial effects [3,4,5,6]. It is especially known for its antioxidant, anti-inflammatory, hepatoprotective, antimicrobial, antispasmodic and antipyretic activity [5,6,7,8]. 

The pharmacological effects of common yarrow are scientifically proven and they are based on the chemical composition of the plant [9,10]. It is stated that the chemical composition of yarrow depends on the region of occurrence; however, studies have shown that the majority of *A. millefolium* L. consists of phenols, sesquiterpenes, coumarins, sterols, dicaffeoylquinic acids [1,11]. The major benefits are ensured by the presence of phenolic compounds such as phenolic acids and flavonoids, which mainly occur as mono- and diglycosides of apigenin, luteolin and quercetin. 

The medicinal applications of *A. millefolium* L. have been known for centuries as it was applied as a treatment of wounds, inflammation and swellings, diarrhea, heartburn, lung diseases, toothache, headache and many more [9,12,13,14]. It also supports the treatment of gastrointestinal disorders, and loss of appetite and relieves menstrual pain. Furthermore, it is used as a mouthwash to promote the healing of cuts [15] and as a component of tea mixtures [10]. 

*Matricaria chamomilla* L. (syn. *M. recutita*, *Chamomilla recutita*) known as German chamomile belongs to the same family of Asteraceae plants and it grows both wild and cultivated in Southern and Eastern Europe, North Africa, West Asia, North and South America and Australia [16]. It is characteristic of its daisy-like flowers, which bloom with a strong aromatic smell from early to midsummer [17]. The plant measures from 15 to 60 cm in height and has branched, erect and smooth stems.

German chamomile is an annual herb cultivated worldwide due to its bioactive compounds and their positive medicinal aspects. According to the studies *M. chamomilla* L. is rich in various terpenoids, flavonoids and lactones, which determines its pharmacological uses [18,19,20,21]. Besides these main groups, it consists of α-bisabolol oxide, camphene, α-pinene, 1,8-cineole, camphor and chamazulene [22]. Previous studies proved the antioxidant, anti-inflammatory, antiphlogistic, anticancer, neuroprotective, antibacterial, anti-allergic, anti-diarrheal and antimicrobial effects [23,24,25,26,27].

According to the data, German chamomile is claimed to be the official drug in pharmacopeia in 26 countries [28]. In traditional medicine, it is used to treat wounds, ulcers, eczema, gout, skin irritations, bruises, burns, sciatica, rheumatic pain, hysteria, nightmares, insomnia and other sleep problems [17,29]. Moreover, it helps to relieve common cold symptoms and to calm nerves and reduce anxiety. In major of cases, it is applied as an infusion of leaves and flowers, but also as essential oils and alcohol extracts [24]. Externally, chamomile is used to treat diaper rash, chicken pox, ear and eye infections, disorders of the eyes including blocked tear ducts, and conjunctivitis [27,30].

The whole plants and their parts, e.g., inflorescences or leaves, can be used as dried material, powders, essential oils or alcohol extracts [31,32,33,34,35,36,37,38,39,40]. Attempts were made to use dried chamomile and yarrow in polymer technology as a component of elastomer mixtures [41,42] as well as an additive for hydrogels [43]. There is no information in the literature on the use of freeze-dried extracts from German chamomile and common yarrow in the technology of elastomeric materials, thus the conducted research constitutes a scientific novelty in this field.

According to the previous studies on the freeze-dried alcohol extracts from plants [44,45], these substances demonstrate the antioxidant and anti-aging potential. Further research indicated the strengthening effect of freeze-dried extracts on the performance properties of vulcanizates based on natural rubber. On this basis and according to the literature, the authors of this manuscript aimed to create elastomer material with improved mechanical strength and increased resistance to external aging factors. It was realized by mixing natural rubber with cross-linking system and freeze-dried alcohol and water extracts from *Matricaria chamomilla* L. and *Achillea millefolium* L. The concept of these studies was introduced due to the environmental issues related to the high usage of petroleum-based polymers and other substances from non-renewable sources. Obtained material may be an alternative to the commonly used composites and contribute to the partial or complete replacement of synthetic polymers, rubbers and additives with renewable raw materials.

## 2. Results

### 2.1. Characteristic of Extracts

#### 2.1.1. Fourier Transform Infrared Spectroscopy (FTIR)

By analyzing obtained spectra, it can be noticed that the absorbance intensity of freeze-dried German chamomile extracts was noticeably higher than the absorbance recorded in the case of yarrow samples, which can be observed from 900 cm^−1^ to 1800 cm^−1^ of wavelength. The highest absorbance in both types of extracts was registered in the case of water-methanol solvents, while the lowest was performed for samples from water. According to these observations, it can be concluded that the selected extract solvent had a significant impact on the composition and properties of the obtained freeze-dried extract. This is due to the different solubility of substances present in extracts in water and alcohol. A detailed description of groups identified according to obtained FTIR spectra is listed in Table 1.

**Figure 1:**
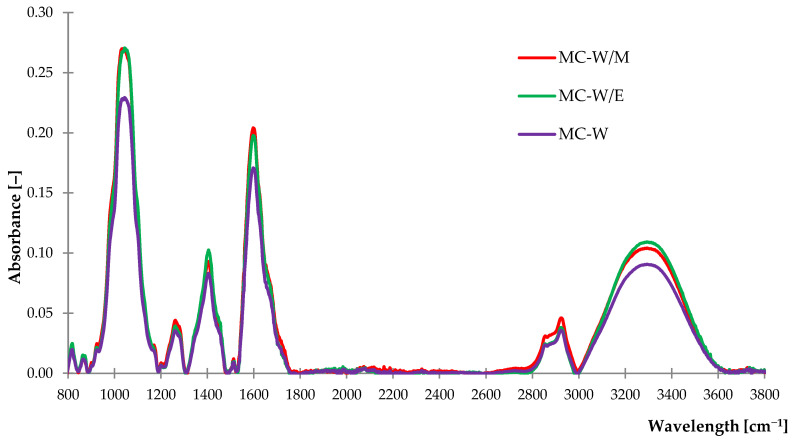
The FTIR spectra of German chamomile freeze-dried extracts.

**Figure 2:**
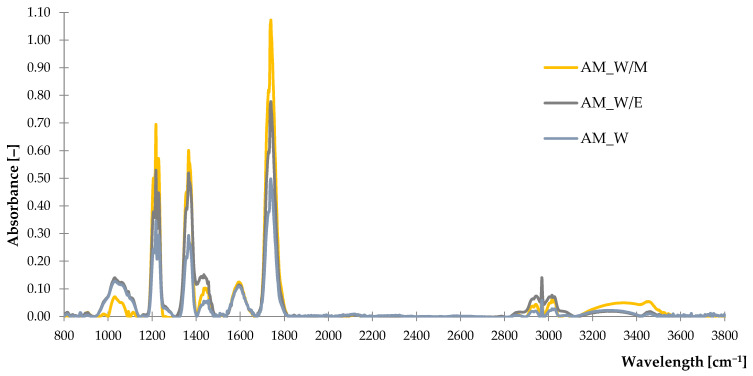
The FTIR spectra of common yarrow freeze-dried extracts.

A broad band from 900 cm^−1^ to 1200 cm^−1^ with a peak in 1030 cm^−1^ recorded at the spectrum (Figure 1) of German chamomile freeze-dried extract is associated with the stretching vibrations of C-O and C-C groups from alcohols, esters, carboxylic acids from tannins, terpenoids, flavonoids and other metabolites [46]. In addition, it refers to the presence of sesquiterpene characteristic for this plant, which is α-bisabolol [47]. Similar groups were identified at the spectrum of *Achillea millefolium* L. (Figure 2), however, the registered peak was less intense. Both examined freeze-dried extracts absorbed light at the wavelength nearby 1250 cm^−1^ and 1400 cm^−1^, where a sample of yarrow presented higher intensity of peaks rather than German chamomile. These results correspond to the vibrations of saturated esters, aliphatic groups and bending vibrations of the O-H bond from phenol groups. Moreover, at 1440 cm^−1^ of the electromagnetic spectrum, stretching vibrations of the C=C bond were recorded, which is related to the aromatic rings from polyphenols and derivatives. A peak registered at 1600 cm^−1^ is related to the vibrations of aliphatic groups and double bonds from the C=C group. These may be correlated with characteristic components of plants: luteolin in German chamomile [46,48] and chamazulene present in both plants [49,50]. A high peak at 1735 cm^−1^ on the spectrum of freeze-dried extract from *Achillea millefolium* L. is associated with C=O bond from carvone and other compounds [50,51]. Absorbance recorded as a band in the range of 2800–2970 cm^−1^ is related to the asymmetric and symmetric vibrations of methyl and methylene groups, while the broad band at 3000–3600 cm^−1^ indicated the presence of O-H stretching vibrations from phenols and alcohols.

Obtained results of FTIR spectra proved the occurrence of bioactive secondary metabolites in freeze-dried extracts from German chamomile and common yarrow. It can be assumed that the antioxidant properties of these substances may reduce the influence of aging factors on vulcanizates based on natural rubber with the addition of freeze-dried extracts.

**Table 1:** Characteristic functional groups registered by FTIR analysis of extracts. Based on Refs. [47,48,49,50,51,52].

Peak Assignments and Type of Vibration	Wave Number [cm^−1^]
*v*(O–H) phenols & alcohols, –C=O_w_ (overtone) and *v*(=C–H_vw_)	3600–3000
*v_s_* (C–H) aliphatic and *v*_as_(–C–H_m_, –CH_3_, –CH_2_)	2970–2800
*v* (C=O)	1730–1690
*v*(C=C) aryl, *d*_vw_(–CH_2_) and (–CH_3_) bending (scissoring) or *v*_vw_(–C–H) bending (rocking)	1600–1500
*sv*(C=C) aromatic	1440
*v*(O–H) bending	1390–1310
*v*(–C–H, –CH_3_)	1372–1337
*v*(–C–C(=O))–O	1250–1160
*v*(–C–O), *v*(–C–H), *v*(–O–H)	1056
*v*_m,vw_(–C–O),	1044/1023
*v*(C–O), (C–C)	1020–1030
*v*(O–C–O) *v*(C–C–C)	1090–1020 900

Abbreviations: *v*—stretching vibrations; *d*—deformation vibrations; *s*—symmetric; *as*—asymmetric; *st*—strong; *w*—weak; *vw*—very weak; *m*—medium; *sv*—skeletal vibration; *a*—axial.

#### 2.1.2. Near-Infrared Spectroscopy (NIR)

**Figure 3:**
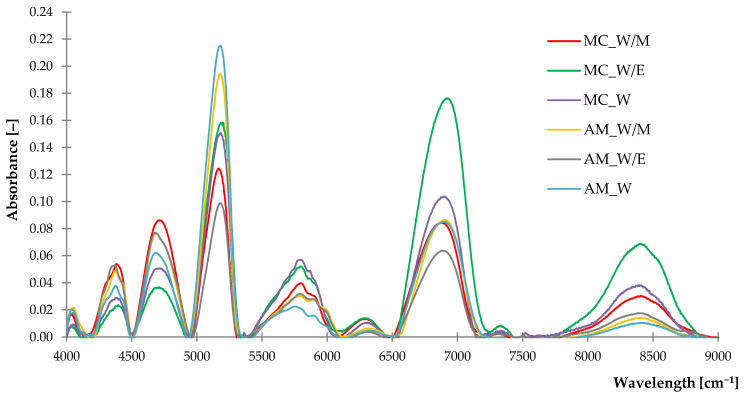
The NIR spectra of freeze-dried extracts.

Investigated extracts demonstrated similar courses of NIR spectra (Figure 3) with slight differences in absorbance along the entire wavelength (4000–9000 cm^−1^). High, intensive peaks recorded at 4531–4890 cm^−1^, 5093–5233 cm^−1^ and 6670–7068 cm^−1^ are probably related to the O-H bond from remaining water and moisture in samples [53]. The rest of the registered peaks were characteristic of stretching vibration of C-H bonds from phenolic compounds [54,55] and carotenoids [56,57,58]. The assignments of identified groups to particular absorbance range is listed in Table 2. Obtained results correspond to the compounds identified by FTIR analysis.

#### 2.1.3. UV-Vis Diffuse Reflectance Spectroscopy

**Figure 4:**
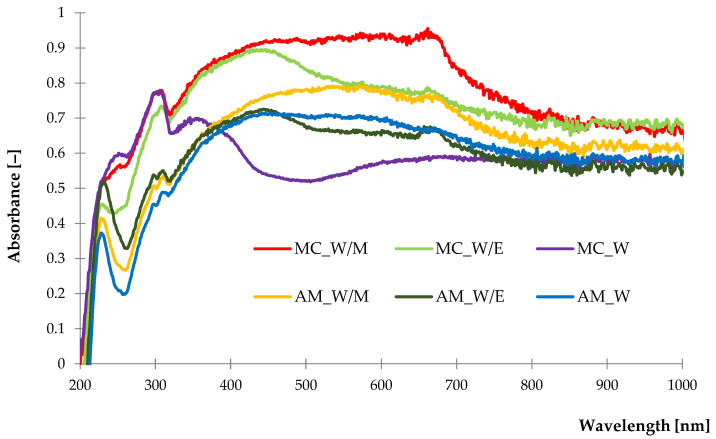
The UV-Vis spectra of freeze-dried extracts.

When analyzing the course of the UV-Vis spectra (Figure 4) recorded for the German chamomile samples, it can be noticed that MC_W/M and MC_W curves were overlapping up to 330 nm, while the MC_W/E sample presented decreased absorption. Over this range, freeze-dried extracts from alcohol-water solutions performed a higher absorbance than from aqueous solvents. In the case of common yarrow freeze-dried extracts, a similar course of spectra was detected regardless of the solvent type and wavelength range.

Peaks recorded at 230–260 nm and 300–350 nm may be associated with the presence of flavonoids, phenols and phenolic acids, according to present data [63]. High absorption recorded at the 380–480 nm and 560–680 nm is characteristic for the chlorophyll “a” and “b”, which probably occurred [64]. A slight increase in absorption was recorded at 670 nm for samples from alcohol-water solvents, which may be related to the higher solubility of chlorophyll in alcohols than in pure water. Moreover, every freeze-dried extract presented high absorbance from 400 nm to 500 nm, which is related to the presence of carotenoids [65].

Identified substances may provide the antioxidant potential of prepared freeze-dried extracts, which correlate with the results obtained by the FTIR and NIR analysis.

#### 2.1.4. Thermal Stability

**Figure 5:**
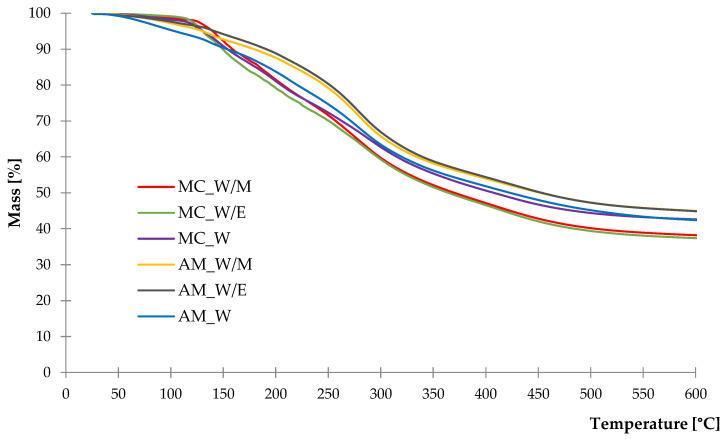
TG curves of freeze-dried extracts.

**Figure 6:**
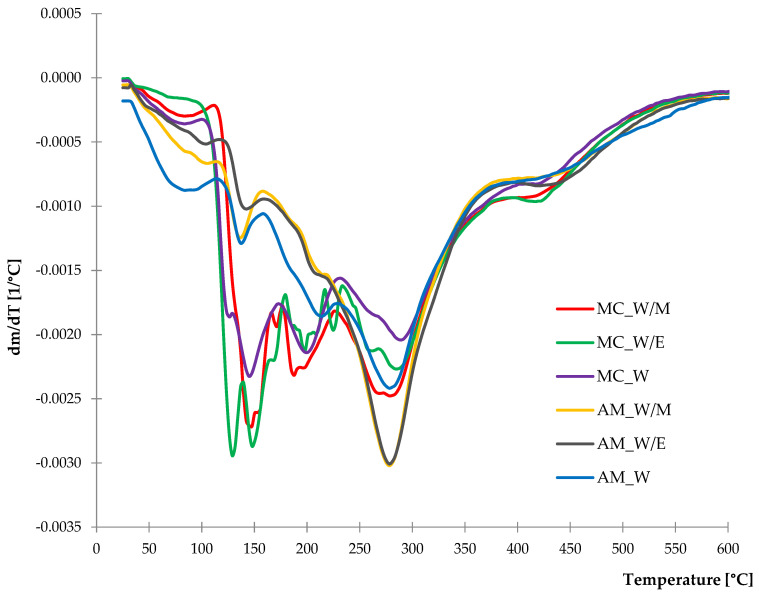
DTG curves of freeze-dried extracts.

As a result of the determined thermogravimetric analysis, both TG and DTG curves were obtained and presented in Figure 5 and Figure 6. Based on TG measurements, it was noticed that majority of the thermal degradation processes occurred from 25 °C to 550 °C leaving the mass residue at 600 °C with ca. 37–45% of starting weight. The residue is the remaining carbon compounds that decompose slowly with increasing temperature beyond the 600 °C. According to the course of DTG curves, it can be observed that the thermal decomposition of samples was a multi-stage process. Percentage mass loss (Δm) in a particular stage was measured and is listed in Table 3.

The course of DTG curves of German chamomile and common yarrow freeze-dried extracts slightly differed. The greater mass loss in the first stage from 25 °C to 120 °C was recorded for *A. millefolium* L. extracts, especially from water solvent. The decrease of mass in this temperature range is related to the evaporation of moisture and remaining solvents from extraction. The next stage can be noticed in the temperature range of 120–170 °C where a rapid and great mass loss was recorded in the case of chamomile extracts in contrast to the yarrow samples. Similar observations were made in the third stage of thermal decomposition, with states for a temperature range of 170–230 °C. From 230 °C to 310 °C, a significant mass loss was registered for the freeze-dried extracts of common yarrow obtained from alcohol-water solvents. Mass loss detected in those stages may be related to the presence of secondary metabolites including phenol compounds, flavonoids, flavanones and others [66,67,68]. The last slight decrease of mass was recorded from 400 °C to 430 °C, which may correspond to carotenoids according to present data [69,70,71].

#### 2.1.5. Total Phenolic Content (TPC) and Antioxidant Activity

The results presented in Table 4 show that *Matricaria chamomilla* L. and *Achillea millefolium* L. extracts contain phenolic compounds and reveal antioxidant properties. TPC values correlated with antioxidant activity. (R = 0.94 when TPC correlated with ABTS values; R = 0.91 when TPC correlated with DPPH values). In authors previous studies, extracts obtained from common nettle and peppermint were investigated [45]. However, chamomile and yarrow extracts contain more phenolic compounds and reveal stronger antioxidant activity.

The results also showed that TPC and antioxidant capacity depend on the extraction solvent used. In these studies, the extraction process was performed using inorganic solvent (water) and organic solvents (methanol and ethanol). For both plants, the highest TPC and the greatest antioxidant potential were detected in water-methanol extracts followed by water-ethanol and water extracts. In addition, extraction efficiency obtained with water was much more lower when compared to organic solvents. Guz et al. [72] also reported that yarrow ethanolic extract showed a higher TPC value and antioxidant activity than water extract. These differences are due to the different solubility of polyphenols in organic and inorganic solvents.

According to literature, *M. chamomilla* L. extract contains hydroxycinnamic acids such as ferulic, chlorogenic, dichlorogenic acid isomers, caffeic, p-coumaric acid, as well as flavonoids such as apigenine and apigenine derivatives (apigenin glucoside, apigenin acetyl glucoside, apigenin glucuronide), quercetin, rutin, rutin trihydrate, kaempferide, luteolin, luteolin glucoside. The dominant compounds are apigenin glucoside, rutin, trihydrate, chlorogenic, p-coumaric acid and ferulic acid [73,74].

The phenolic composition of *A. millefolium* L. extracts has been poorly investigated in the literature. In yarrow, the following phenolic acids and their derivatives were detected: gallic, caffeic acids, chlorogenic acid derivative, protocatechuic acid hexoside. Among flavonoids, quercetin, quercetin rutinoside, quercetin glucoside, luteolin, luteolin glucoside, kaempferol, myricetin, apigenin, apigenin glucoside were found [75,76].

### 2.2. Characteristic of Vulcanizates

#### 2.2.1. Rheometric Properties

Rheometric measurements were conducted to provide several valid parameters, which enabled planning and performing the vulcanization process of rubber mixtures and indirectly predicting the cross-linking density of vulcanized mixtures. The first defined parameter was the optimal vulcanization time (t_90_), which is the time required for the torque to reach 90% of the maximum value [77]. An increase in torque (ΔM) was a second parameter based on the difference between the maximum (M_max_) and minimum (M_min_) torque gained during the exam [78].

**Figure 7:**
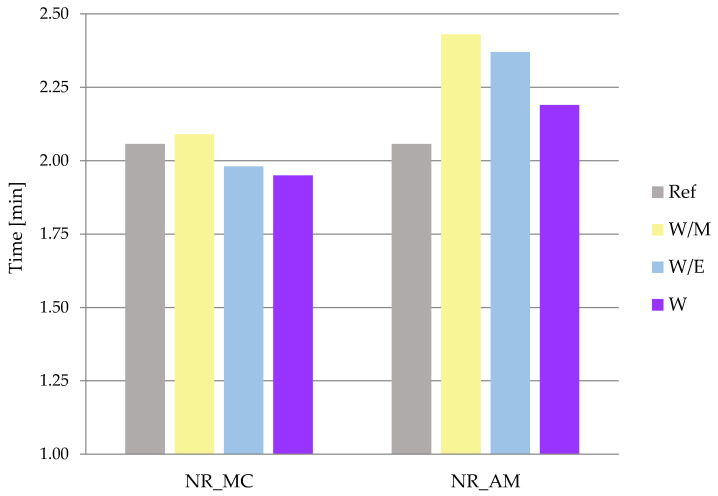
Optimal vulcanization time (t_90_) of vulcanizates.

In the case of NR_MC extracts from water and water-ethanol solvents, the optimal vulcanization time (Figure 7) slightly decreased compared to the reference sample. The t_90_ value of German chamomile freeze-dried extract from water-methanol was 2.09 min, which was comparable to the measurement for reference vulcanizate (t_90_ = 2.07 min). In contrast, the addition of *A. millefolium* L. extracts to rubber mixtures caused a noticeable increase in optimal vulcanization time, which ranged from 2.19 min for NR_AM_W to 2.43 min for NR_AM_W/M.

Differences in optimal vulcanization time between both types of vulcanizates with bio-additives is probably related to the thermal stability of added extracts. According to the thermogravimetric analysis, extract from German chamomile demonstrated lower thermal stability compared to the common yarrow up to the temperature of 160 °C, which was the temperature of rheometric measurements. On this basis, it can be concluded that the increase in optimal vulcanization time was related to the organic phase of added extracts and remaining secondary metabolites in prepared vulcanizates. These may have caused the decrease in the activation and action of the curing system compounds.

**Figure 8:**
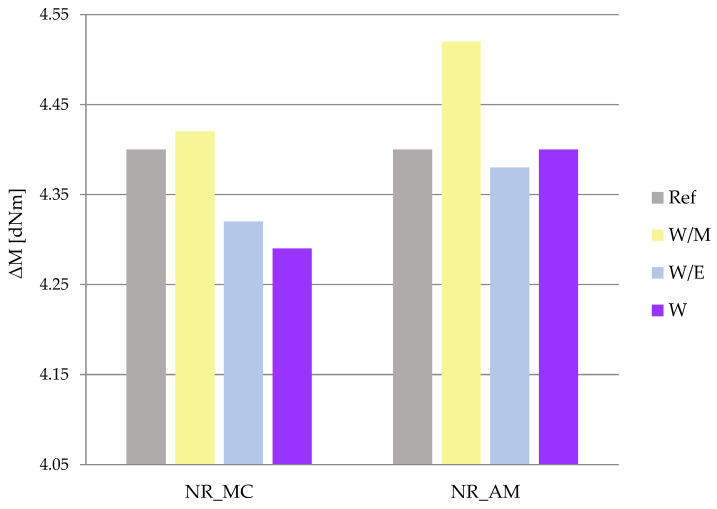
Increase in torque (ΔM).

The increase in torque (ΔM = M_max_ − M_min_) is considered to be the indirect measurement of cross-linking density as the M_min_ is the lowest value of torque indicated on the vulcanization curve, equal to the viscosity of the sample in this particular moment, while M_max_ is the highest value of torque indicated on the vulcanization curve, which characterizes the vulcanizates stiffness at the end of vulcanization process [79]. Obtained results (Figure 8) may indicate increased cross-linking density in the case of rubber mixtures with extracts prepared in a water-methanol solvent. The rest of the samples gained decreased values of ΔM compared to the reference sample, which may be related to the lower cross-linking density of vulcanizates with the addition of water and water-ethanol extracts from both chamomile and yarrow.

#### 2.2.2. Cross-Linking Density

During the exploitation of elastomer and polymer products, various processes of aging occur, which have a direct influence on the properties of the material and as a consequence also on its lifetime. Since it is possible to carry out the simulated aging of polymer materials, obtained vulcanizates were subjected to UV radiation and elevated temperature to predict the influence of aging factors on the cross-linking density, mechanical properties and change of color.

Aging factors can cause either the degradation process of elastomer material or it is further cross-linking known as the post-curing [80]. Post-curing occurs due to the recombination of free radicals into branched structures that expand the internal spatial network of the polymer. Moreover, it can be related to the influence of elevated temperature or UV radiation, which may lead to the activation of the remaining cross-linking system compounds used in the rubber mixture and also contribute to the formation of new network nodes [80,81]. On the other hand, elastomer mixtures based on natural rubber are more likely to degrade due to the uncontrolled and irreversible changes in cis-1,4-polyisoprene structure including chain scissions and the formation of chain molecules, which leads to the reduction of elastomer molecular weight. As the consequence, created entangled chains may reduce the mobility of the molecules and thus the flexibility of the vulcanizate, leading to decreased swelling and increased cross-linking density.

By analyzing the values of cross-linking density γ_e_ (Table 5) of unaged vulcanizates, it was observed that the addition of extracts from water-methanol solutions caused the increase in cross-linking density compared to the reference sample, which correlates with the results of ΔM from rheometric measurements. The highest γ_e_ between 1.92 × 10^−5^ mol/cm^3^ and 1.94 × 10^−5^ mol/cm^3^ was obtained for German chamomile samples both aged and unaged, though in the case of NR_AM vulcanizates cross-linking density was likewise high. The tendency of the rest bio-additives is quite the opposite. Regardless of the type of used extract, cross-linking density was lower than the reference sample and ranged between 1.31 × 10^−5^ mol/cm^3^ in the case of NR_AM_W to 1.60 × 10^−5^ mol/cm^3^ for NR_MC_W/E. Aging factors probably caused the post-curing of the reference vulcanizate as it increased from 1.63 × 10^−5^ mol/cm^3^ to 1.84 × 10^−5^ mol/cm^3^ after ultraviolet radiation and to 1.82 × 10^−5^ mol/cm^3^ after the thermo-oxidative process. Slight changes in the values of γ_e_ parameter were observed in the case of mixtures with the addition of freeze-dried extracts after both types of simulated aging. This may indicate the beneficial influence of secondary metabolites present in extracts, which protect materials from the influence of aging factors. According to literature, phenols and derivatives may act as reducing agents (free radical terminators), metal chelators and singlet oxygen quenchers [82].

#### 2.2.3. Mechanical Properties

The influence of aging factors on the mechanical properties of elastomer vulcanizates was established by the examination of the tensile strength (TS) and elongation at break (Eb). Moreover, the aging factor (K) was determined according to the TS and Eb results.

**Figure 9:**
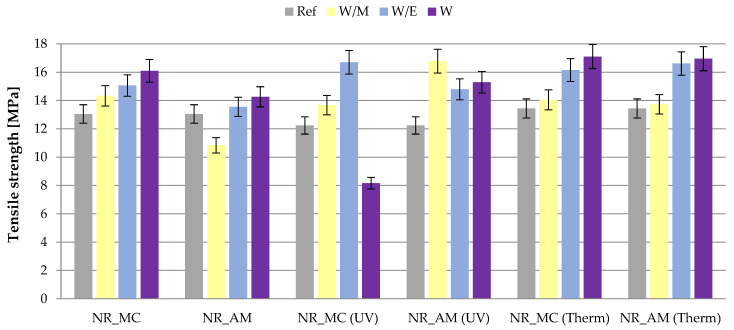
Results of tensile strength measurements of unaged samples, after ultraviolet (UV) and thermo-oxidative (Therm) aging.

The tensile strength (Figure 9) of the unaged reference system was equal to 13.05 MPa. The addition of German chamomile extract caused the improvement of vulcanizate’s mechanical strength as the TS value reached 14.33 MPa for the NR_MC_W/M, 15.06 MPa for NR_MC_W/E and 16.09 MPa for NR_MC_W. A similar tendency was observed for the common yarrow extracts, although the improvement was slighter (13.55 MPa for NR_AM_W/E; 14.26 MPa for NR_AM_W) and in the case of extract from water-methanol solvent tensile strength was decreased compared to the reference sample.

Ultraviolet radiation caused deterioration of mechanical properties of reference vulcanizate and NR_MC_W compared to unaged ones. In the rest samples, UV and thermo-oxidative aging contributed to the significant increment of tensile strength. The highest results were obtained for the vulcanizates with extracts from water-ethanol and water solvents after the thermo-oxidative aging. The tensile strength in those cases reached 16.15 MPa to 17.10 MPa.

The elongation at break (Table 6) of the unaged reference vulcanizate was higher compared to the rest of the samples, while after aging processes the opposite effect was observed. Obtained results corresponded to calculated cross-linking densities of prepared samples, which indicate the variety in the elasticity of vulcanizates.

Deterioration of mechanical strength despite the increased cross-linking may be related to the achievement of the so-called critical limit of cross-linking density, beyond which the mechanical properties of vulcanizates begin to deteriorate [83].

**Figure 10:**
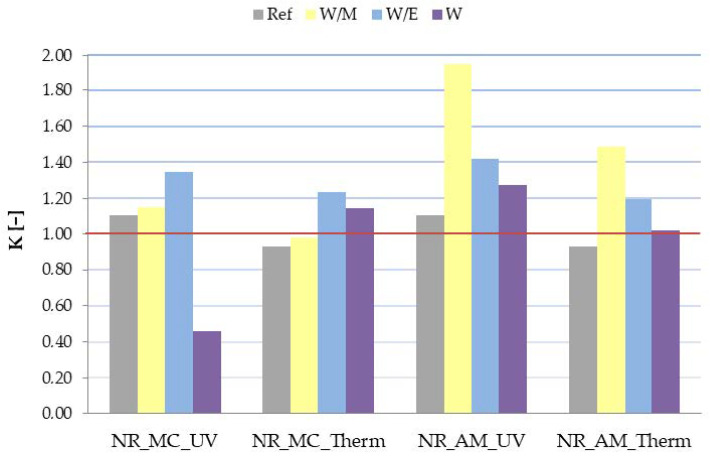
Aging factor (K) of samples after ultraviolet (UV) and thermo-oxidative (Therm) aging.

When analyzing the aging factor (K) it can be assumed that the material indicated the increase of mechanical properties after aging, when the value of K exceeds 1 (orange line on Figure 10). According to obtained results (Figure 10), it can be stated that in general the addition of freeze-dried extracts from *M. chamomilla* L. and *A. millefolium* L. caused a significant improvement in mechanical properties. The only exception was observed for vulcanizates NR_MC_W after ultraviolet degradation and NR_MC_W/M after the thermo-oxidative process, where K is lower than 1. The greatest increment was established for NR_AM_W/M samples in both types of aging. Satisfying results were also gained for all types of vulcanizates with the addition of natural extract from a water-ethanol solution. The aging factor in those cases reached 1.20–1.42.

Based on TS, Eb and K measurements, it can be stated that the addition of freeze-dried extracts from German chamomile and common yarrow to the rubber mixtures contributed to the improved resistance to the external factors and as a consequence increment of mechanical properties.

#### 2.2.4. Color Stability

The color change test was conducted to provide the information about the influence of the aging factor on the external appearance of samples.

**Figure 11:**
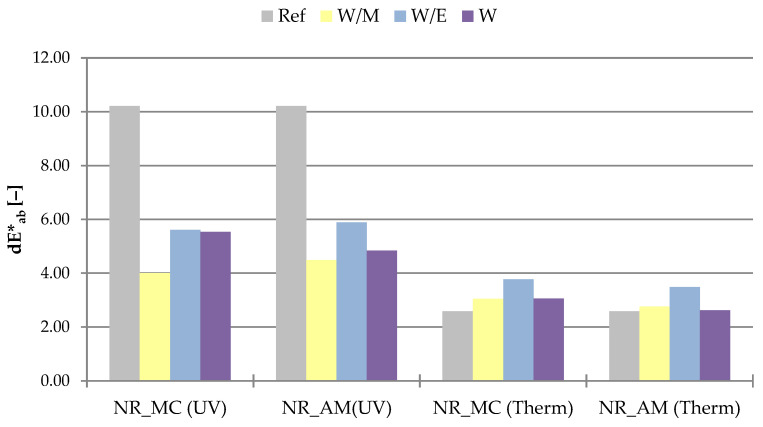
The color change parameter (dE*_ab_) of samples after ultraviolet (UV) and thermo-oxidative (Therm) aging.

The results of measured color change dE*_ab_ were presented as a bar graph in the Figure 11.

The greater color change was observed in the case of UV aging with a maximum value of 10.21 for the reference system. Vulcanizates with the addition of natural extracts showed a decreased change of color reaching from 4.00 (NR_MC_W/M) to 5.89 (NR_AM_W/E). The reduced dE*_ab_ values may be related to the presence of antioxidant secondary metabolites in extracts. These compounds can react with free radicals in various ways including hydrogen atoms and single electron transfers or transition metal chelation [84]. According to this, added extracts could prevent elastomer vulcanizates from the degrading influence of UV radiation on their surface.

In contrast, thermo-oxidative aging caused less color change of reference vulcanizate compared to the rest of the samples, although the differences were slightly noticeable. Observed color changes ranged from 2.58 to 3.77, which according to data are changes imperceptible to the human eye [85].

#### 2.2.5. Barrier Properties

**Figure 12:**
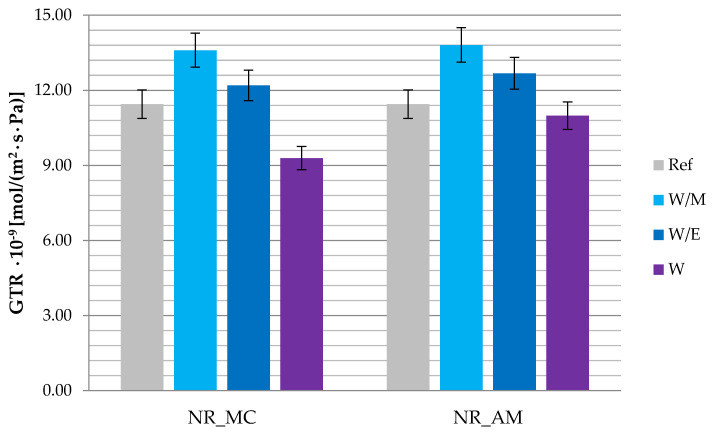
The gas transmission rate (GTR) values of tested vulcanizates.

The gas transmission rate (GTR) was measured to establish the barrier properties of materials containing freeze-dried extracts and the reference system. Vulcanizates with the addition of extracts from water-methanol and water-ethanol solvents (Figure 12) presented increased values of GTR parameter reaching 12.20–13.81·10^−9^ mol/(m^2^·s·Pa), while the gas transmission rate for the reference sample was 11.44·10^−9^ mol/(m^2^·s·Pa). The lowest gas permeability below 11·10^−9^ mol/(m^2^·s·Pa) was established in the case of water extracts from both German chamomile and common yarrow.

## 3. Materials and Methods

### 3.1. Materials

- Elastomer matrix: natural rubber RSS I (NR) provided by Torimex Chemicals Sp. z.o.o. (Konstantynów Łódzki, Poland);
- Cross-linking system: sulphur (Siarkopol, Tarnobrzeg, Poland), stearin (POCH, Gliwice, Poland), 2-mercaptobenzothiazole (MBT) (Sigma-Aldrich, Poznań, Poland), micro-sized zinc oxide (ZnO) (Huta Będzin, Poland);
- Dried plants: *Matricaria chamomilla* L. and *Achillea millefolium* L. provided by Ziołowy Zakątek (Koryciny, Poland);
- Both plants used for this study was purchased from certificated food company. Leaves of plants were harvested in the area of Podlaskie Voivodeship (Poland) before the flowering. Dried leaves were packed into paper bags. Products were ground in food chopper to obtain powders.

### 3.2. Preparation of Freeze-Dried Extracts

Three different solvents were prepared to perform extraction: pure water (W); methanol-water (W/M) and ethanol-water (W/E) in 50/50 volume. The 15 g of dried and ground plant material was placed in a cellulose thimble and immersed in a selected solvent (100 mL). Performed extraction was a three-stage process conducted in a Series 148 extractor from Velp (Usmate Velate, Italy). The prepared mixture of solvent and plant material was brought to a boiling temperature and the extraction process was carried out for 1.5 h. Afterward, the thimbles were lifted over the solvent and rinsed with the evaporating solvent (step 1). Then, another 15 g portion of plant material was added to the new thimble and the beaker with obtained extract was filled with solvent to the volume of 100 mL. The thimbles were immersed again for another 1.5 h from the reflux point. Further steps were identical to step 1 (step 2). In step 3, the thimble was changed for the last time. After the extraction, the solvent was evaporated in a rotary evaporator Heidolph, Laborata 4001 (Teltow, Germany) to the volume of ca. 40 mL.

To obtain products in solid form, extracts were subjected to the freeze-drying process in Labconco Freezzone 2.5 Plus (Kansas City, MN, USA).

Following abbreviations were used to identify obtained samples:

- MC-W/M—freeze-dried extract of *M. chamomilla* L. from water-methanol solvent;
- MC-W/E—freeze-dried extract of *M. chamomilla* L. from water-ethanol solvent;
- MC-W—freeze-dried extract of *M. chamomilla* L. from water solvent;
- AM-W/M—freeze-dried extract of *A. millefolium* L. from water-methanol solvent;
- AM-W/E—freeze-dried extract of *A. millefolium* L. from water-ethanol solvent;
- AM-W—freeze-dried extract of *A. millefolium* L. from water solvent.

### 3.3. Preparation of Rubber Mixtures and Vulcanizates

In the first stage, natural rubber was plastified in Brabender measuring mixer N50 (Brabender Technologie GmBH & Co. KG, Duisburg, Germany) for 4 min with a rotational speed of 40 rpm and a temperature range of 40–60 °C. Then, the freeze-dried extract was added under the same conditions. The next step was performed in a two-roll mill at room temperature by mixing natural rubber with the addition of freeze-dried extract with cross-linking system. The same procedure was used to prepare a separate mixture without the addition of freeze-dried extracts as a reference sample.

The composition of prepared rubber mixtures are presented in Table 7.

The vulcanization process of rubber mixtures was performed in steel molds placed between the shelves of an electrically heated hydraulic press. Prepared mixtures were vulcanized at the temperature of 160 °C and at 15 MPa pressure for curing time determined from rheometric measurements.

### 3.4. Freeze-Dried Extracts Characterization Methods

FTIR spectra were recorded using Nicolet 6700 spectrophotometer (Thermo Fischer Scientific Instruments, Waltham, MA, USA) equipped with a diamond adapter (Smart Orbit ATR sampling accessory). The analysis was performed with 128 scans over the range of 800–3800 cm^−1^.

The Evolution 201/220 UV–Visible Spectrophotometer (Thermo Scientific, Waltham, MA, USA) was used to obtain UV–Vis spectra. The device was equipped with xenon, tungsten and deuterium lamps as a source of light. Materials were placed in the glass cuvettes. Measurements of the natural extracts were conducted in the spectral range of 1000 to 200 nm in the “Scan” mode (measurement of the light passing through the sample over the entire spectral range).

Near-infrared spectroscopy technique was used to identify bonds characteristic of freeze-dried extracts structure in the electromagnetic spectrum of 9000–4000 cm^−1^. For the analysis, the Nicolet 6700 spectrophotometer (Thermo Fischer Scientific Instruments, Waltham, MA, USA) was used. Spectra were recorded in absorption mode of 128 scans and 8 cm^−1^ resolution.

The thermal stability was examined using the TGA/DSC1 analyzer (Mettler Toledo, Columbus, OH, USA/Greifensee, Switzerland). The analysis was conducted in the temperature range of 25 to 600 °C with a 10 °C/min heating rate in a flow of nitrogen at 60 mL/min.

The total phenolic content (TPC) of extracts was determined using the Folin–Ciocalteu method. The reaction contained: 100 µL of the extract; 200 µL of Folin–Ciocalteu reagent; 1 mL of 20% Na_2_CO_3_ solution; and 2 mL of distilled water. The blank sample contained 100 µL of distilled water instead of the extract. The samples were mixed and stored at room temperature in a dark place for 1 h. The absorbance was measured at the wavelength of 765 nm against the blank sample using a Cecil CE2041 spectrophotometer (Cecil Instruments Limited, Cambridge, UK). A calibration curve for gallic acid was prepared. The TPC was quantified according to a calibration curve and expressed as gallic acid equivalents (µgGAE/mL).

The antioxidant activity of the extracts was measured with two free radical scavenging methods, DPPH and ABTS, as it was previously described by Efenberger-Szmechtyk et al. [86].

- DPPH

A total of 2.4. mg of 2,2-diphenyl-1-picrylhydrazyl (DPPH) (Sigma-Aldrich, St Louis, MO, USA) was dissolved with 100 mL of 80% ethanol. The absorbance of DPPH solution was adjusted to approximately 0.900 at 515 nm. A total of 50 µL of the extract was added to 1.95 mL of the DPPH free radical solution. The samples were kept for 15 min at room temperature in a dark place. The absorbance was measured at the wavelength of 515 nm against 80% ethanol using a Cecil CE2041 spectrophotometer (Cecil Instruments Limited, Cambridge, UK). A calibration curve was prepared for Trolox (6-hydroxy-2,5,7,8- tetramethylchroman-2-carboxylic acid) (Sigma-Aldrich, St Louis, MO, USA). The antioxidant activity of the extract was expressed as Trolox equivalents (mgTE/mL).

- ABTS

ABTS (2,20-azinobis(3-ethylbenzothiazoline-6-sulphonic acid) (Sigma-Aldrich, St Louis, MO, USA) was dissolved in water to obtain a concentration equal to 7 mmol. K_2_S_2_O_8_ was added to ABTS stock solution to obtain a final concentration of 2.45 mmol. The solution was stored for 12–16 h in a dark place at room temperature to obtain ABTS radical cation (ABTS+). The ABTS+ solution was diluted to reach an absorbance of 0.700 at 734 nm. A total of 30 µL of the extract was added to 3 mL of ABTS+ solution. The samples were stored for 10 min and the absorbance was measured at the wavelength of 734 nm against distilled water using a Cecil CE2041 spectrophotometer (Cecil Instruments Limited). The antioxidant activity was calculated based on a calibration curve prepared for Trolox and expressed as Trolox equivalents (mgTE/mL).

Mean values, standard deviations (SD) and Pearson’s correlation coefficient R were calculated using Microsoft Excel 2019. Tukey’s honestly significant differences (HSD) test (*p* < 0.05) was performed using R 3.4.0 (R Core Team, Vienna, Austria) to assess statistically significant differences between samples.

### 3.5. Vulcanizates Characterization Methods

Rheometric properties of rubber mixtures were determined as follows: samples were placed in the measuring chamber of the MonTech DRPA 300 Rheometer (MonTech Werkstoffprüfmaschinen GmbH, Buchen, Germany) and subjected to changes in the torque of the oscillating disc as a function of time in 160 °C. During tests, two parameters were measured: optimal curing time (t_90_) and increase in torque (ΔM). Merck (1000 mg/L) standard solutions were prepared for calibration curves.

Vulcanizates were subjected to the accelerated simulation of thermo-oxidative and ultraviolet aging. Thermo-oxidative simulation was performed in a forced air dryer Binder Model FED 56 (BINDER GmbH, Tuttlingen, Germany) at 70 °C for 14 days. The ultraviolet process was carried out in the UV chamber of Atlas UV 2000 (ATLAS Material Testing Technology GmbH, Duisburg, Germany). The UV chamber was set in the following conditions: the day and night segment: 0.78 W/m^2^; temperature: 60 °C; duration: 72 h.

Non-aged and aged vulcanizates were tested for the cross-linking density of the spatial network. Examinations were conducted according to the solvent-swelling measurements in toluene. Results were calculated from the Flory–Rehner Equation (1) [87]:

(1) $$\gamma_{e}\;=\;\frac{\ln\left({1\;-\;V}_{r}\right){\;+\;V}_{r}{\;+\;\mu V}_{r}^{2}}{V_{0}\left(V_{r}^{\frac{1}{3}}\;-\;\frac{V_{r}}{2}\right)}\;$$

where γ_e_—the cross-linking density (mol/cm^3^), V_0_—the molecular volume of solvent (106.7 cm^3^/mol), μ—the Huggins parameter of the NR-solvent interaction calculated from Equation (2):

(2) $${\mu \;=\;\mu }_{0}\;+\;\beta \cdot V_{r}$$

where μ_0_—the parameter connected with non-cross-linked/solvent, β—the constant consideration of the impact of cross-linking on parameter polymer/solvent, natural rubber–toluene interaction factor μ_0_ and β were experimentally (μ_0_ = 0.478, β = 0.228); V_r_—the volume fraction of elastomer in the swollen gel (Equation (3)):

(3) $$V_{r}\;=\;\frac{1}{{1\;+\;Q}_{w}\frac{Q_{k}}{Q_{r}}}$$

where Q_w_—weight of equilibrium swelling, Q_k_—density of rubber (g/cm^3^) (0.99 g/cm^3^), Q_r_—density of solvent (g/cm^3^) (0.86 g/cm^3^).

Barrier properties were investigated by using manometric method. Tests were performed according to the ASTM standard D1434 based od through-plane air permeability and the gas transmission rate (GTR) was calculated from the following Equation (4) [88]:

(4) $$\mathrm{GTR}\;=\;\frac{V_{c}}{R\cdot T\cdot P_{u}\cdot A}\cdot \frac{\mathrm{dp}}{\mathrm{dt}}$$

where V_c_—the volume of the low-pressure chamber (L), T—temperature (K), P_u_—the gas pressure in the high-pressure chamber (Pa), A—area permeation of gas through the sample (m^2^), dp/dt—pressure changes per unit time (Pa/s), R—gas constant 8.31 × 10^3^ ((L·Pa)/(K·mol)).

Dumbbell-shaped samples of aged and non-aged vulcanizates were examined according to ISO-37 using a static material testing machine Zwick (model 1435, Ulm, Germany). Tests were conducted at room temperature and at the cross-head speed of 500 mm/min. Measurements were performed to determine tensile strength (TS), elongation at break (E_b_) and aging coefficient (K) according to the following Equation (5) [89]:

(5) $$K\;=\;\frac{{(\mathrm{TS}\cdot E_{b})}_{\mathrm{after}\;\mathrm{aging}}}{{(\mathrm{TS}\cdot E_{b})}_{\mathrm{before}\;\mathrm{aging}}}$$

Aged and non-aged elastomer vulcanizates were tested according to the PN-EN ISO 105-J01 standard using the Konica Minolta CM-3600d spectrophotometer (Sony, Tokyo, Japan) to analyze the influence of degradation factors on the color stability of samples. The measurements were conducted in the spectral range of 360–740 nm. The total color change (dE*_ab_) was determined according to the CIE-Lab color space from Equation (6) [90]:

(6) $${\mathrm{dE}}_{\mathrm{ab}}^{*}\;=\;\sqrt{∆a^{2}\;+\;∆b^{2}\;+\;∆L^{2}}$$

where Δa—deviation from the color of the reference sample in the axis of red–green; Δb—deviation from the color of the reference sample in the axis of yellow–blue, ΔL—deviation in brightness parameter from the color of the reference sample.

## 4. Conclusions

The spectroscopic analyses (FTIR, NIR, UV-Vis) proved that freeze-dried extracts from *Matricaria chamomilla* L. and *Achillea millefolium* L. contained secondary metabolites, such as phenolic acids, flavonoids, flavanones, carotenoids, terpenoids and others. The antioxidant potential resulting from the presence of these compounds was confirmed by the studies of total phenolic content (TPLC) and antioxidant activity by DPPH and ABTS methods. The greatest antioxidant character was established for extracts obtained from water-methanol and water-ethanol solvents. Based on the conducted thermogravimetric analysis, it can be concluded that extracts from common yarrow are more thermal stable compared to the German chamomile as the percentage mass loss up to 230 °C was significantly lower for those samples.

This might have affected the behavior of rubber mixtures during the rheometric measurements. According to the results, the optimal vulcanization time (t_90_) was noticeably lower in the case of mixtures containing extract of German chamomile, while samples with the addition of common yarrow freeze-dried extract presented an extension of the t_90_ parameter. The highest values of increase in torque were established for the vulcanizates containing water-methanol extracts, which corresponded to the results obtained by the cross-linking density measurement (γ_e_). The γ_e_ parameter for the rest of examined samples was decreased compared to the reference sample, which may be a result of the action of phenol compounds as reducing agents, metal chelators or singlet oxygen quenchers. On the other hand, gas transmission rate (GTR) was increased after the addition of water-methanol and water-ethanol freeze-dried extracts, which was not observed in the case of vulcanizates containing water extracts. Studies of the tensile strength (TS) and elongation at break (Eb) proved the beneficial influence of freeze-dried extracts on the mechanical properties of prepared vulcanizates. The results of TS, Eb and aging factor (K) showed that extracts provided additional resistance to the aging factors, which was confirmed by the study of color change.

According to the presented results, the addition of freeze-dried extracts from German chamomile and common yarrow to natural rubber mixtures had a significant impact on the properties of vulcanizates. The improved resistance to external aging factors and increment of mechanical properties are the main indicators of their beneficial influence on elastomer materials.

The total phenolic content of examined freeze-dried extracts was nearly two times higher compared to the results from previous research studies on freeze-dried extracts from peppermint and common nettle [45]. It was proved by FTIR analysis, where the intensity of absorbance was significantly increased in the case of common yarrow, which may be related to the increased content of secondary metabolites. A similar tendency was observed by the analysis of mechanical properties. The maximum tensile strength registered for aged samples of vulcanizates with the addition of freeze-dried extracts from German chamomile and common yarrow ranged from 14.05 MPa to 17.10 MPa, while the previous manuscript showed the maximum of 14.33 MPa in the case of vulcanizates with the addition of freeze-dried extract from peppermint after thermo-oxidative aging.

To sum up, freeze-dried extracts from *Matricaria chamomilla* L. and *Achillea millefolium* L. proved their increased antioxidant activity and strengthening effect on vulcanizates compared to previous studies on *Mentha piperita* L. and *Urtica dioica* L. These extracts may be used as a natural renewable additive to elastomer vulcanizates and as a consequence improve their properties and extend their lifetime.

## Figures and Tables

**Table 2:** Characteristic functional groups registered by NIR analysis. Based on Refs. [59,60,61,62].

Peak Assignments and Type of Vibration	Wavelength [cm^−1^]
*s*(–CH_n_)	4034–4040
*s*(–C–H)	4375–4408
*s*(–O–H)	4531–4890
*s*(–O–H)	5093–5233
*s*(–C–H) first overtone	5551–5928
*s*(–O–H)	6670–7068
*s*(–C–H) second overtone	8093–8550

Abbreviations: *s*—stretching.

**Table 3:** Percentage mass losses (Δm) of samples in various temperature ranges from 25 to 600 °C and residue after thermal decomposition at 600 °C (R_600_).

Sample	Δm_25–120 °C_ (%)	Δm_120–170 °C_ (%)	Δm_170–230 °C_ (%)	Δm_230–310 °C_ (%)	Δm_310–600 °C_ (%)	R_600_ (%)
MC_W/M	1.93	10.04	12.54	17.70	19.58	38.21
MC_W/E	2.40	12.37	11.76	16.16	19.91	37.40
MC_W	3.12	9.78	11.55	14.69	18.26	42.60
AM_W/M	4.15	4.83	8.06	19.25	18.74	44.97
AM_W/E	3.38	4.23	8.27	19.35	19.89	44.88
AM_W	6.36	5.31	10.03	16.72	19.18	42.40

**Table 4:** Total phenolic content and antioxidant activity of *Matricaria chamomilla* L. and *Achillea millefolium* L. leaf extracts. The results are expressed as mean ± SD.

Sample	TPC (µg_GAE_/mL)	Antioxidant Activity (mg_TE_/mL)	
		ABTS	DPPH
MC_W/M	14,207.8 ± 39.2 ^b^	11.82 ± 0.11 ^a^	23.57 ± 0.29 ^b^
MC_W/E	8327.6 ± 177.7 ^c^	10.88 ± 0.14 ^b^	24.21 ± 0.22 ^a^
MC_W	1038.2 ± 30.0 ^d^	1.68 ± 0.03 ^d^	2.39 ± 0.01 ^d^
AM_W/M	15,881.8 ± 606.1 ^a^	11.49 ± 0.02 ^a^	23.28 ± 0.27 ^b^
AM_W/E	7961.8 ± 282.2 ^c^	7.75 ± 0.20 ^c^	15.76 ± 0.90 ^c^
AM_W	276.8 ± 19.6 ^e^	0.48 ± 0.03 ^e^	1.16 ± 0.14 ^e^

^a,b,c,d,e^—statistically significant differences between samples (*p* < 0.05).

**Table 5:** Results of the cross-linking density (γ_e_) before degradation process (Unaged) and after ultraviolet (UV) and thermo-oxidative (Therm) aging.

Sample	γ_e_ × 10^−5^ (mol/cm^3^)		
	Unaged	UV	Therm
Ref	1.63 ± 0.02	1.84 ± 0.03	1.82 ± 0.03
NR_MC_W/M	1.93 ± 0.03	1.92 ± 0.04	1.94 ± 0.02
NR_MC_W/E	1.60 ± 0.03	1.65 ± 0.03	1.75 ± 0.03
NR_MC_W	1.52 ± 0.04	1.62 ± 0.02	1.41 ± 0.01
NR_AM_W/M	1.87 ± 0.03	1.88 ± 0.01	1.92 ± 0.04
NR_AM_W/E	1.46 ± 0.04	1.54 ± 0.03	1.51 ± 0.02
NR_AM_W	1.31 ± 0.02	1.50 ± 0.03	1.40 ± 0.04

**Table 6:** Elongation at break before (Eb) and after ultraviolet (Eb_UV_) and thermo-oxidative (Eb_Therm_) aging.

Sample	Eb (%)	Eb_UV_ (%)	Eb_Therm_ (%)
Ref	663.43 ± 1.59	586.59 ± 4.80	584.68 ± 6.24
NR_MC_W/M	617.75 ± 3.32	782.24 ± 2.25	626.25 ± 5.50
NR_MC_W/E	644.19 ± 4.25	784.10 ± 3.23	743.91 ± 4.57
NR_MC_W	646.01 ± 5.37	756.13 ± 2.55	697.56 ± 3.49
NR_AM_W/M	584.85 ± 4.88	760.09 ± 5.13	754.37 ± 4.12
NR_AM_W/E	627.80 ± 6.01	788.24 ± 3.99	570.92 ± 5.20
NR_AM_W	650.20 ± 4.37	772.37 ± 2.47	558.08 ± 3.56

**Table 7:** Formulations of rubber mixtures.

Sample Name	Extract	NR	Stearin	ZnO	MBT	Sulfur
	(phr ^1^)					
Reference Sample (Ref)	0	100	1	5	2	2
NR-MC-W/M ^2^	5.0	100	1	5	2	2
NR-MC-W/E ^3^						
NR-MC-W ^4^						
NR-AM-W/M ^5^						
NR-AM-W/E ^6^						
NR-AM-W ^7^						

^1^ phr–parts per hundred parts of rubber; ^2^ NR-MC-W/M—vulcanizates with addition of freeze–dried extract of German chamomile from water–methanol solution; ^3^ NR-MC-W/E—vulcanizates with addition of freeze–dried extract of German chamomile from water–ethanol solution; ^4^ NR-MC-W—vulcanizates with addition of freeze–dried extract of German chamomile from water solution; ^5^ NR-AM-W/M—vulcanizates with addition of freeze—dried extract of yarrow from water—methanol solution; ^6^ NR-AM-W/E—vulcanizates with addition of freeze–dried extract of yarrow from water–ethanol solution; ^7^ NR-AM-W—vulcanizates with addition of freeze–dried extract of yarrow from water solution.

## Data Availability

Data sharing not applicable.
